# Dietary phytase effects on copper requirements of broilers

**DOI:** 10.3389/fvets.2023.1170488

**Published:** 2023-09-07

**Authors:** Patricia Soster, Sergio Luiz Vieira, Julmar Costa Feijo, Walter Edmundo Altevogt, Giovane B. Tormes

**Affiliations:** Department of Animal Science, Federal University of Rio Grande do Sul, Porto Alegre, Brazil

**Keywords:** broiler, copper, phytase, mineral, requirement

## Abstract

Information on the availability of Cu from plant feedstuffs for broilers in the presence of phytase is scarce. The present research has been conducted with the objective of evaluating the Cu requirements of broilers when fed corn-soy diets with or without phytase. A total of 640 one-day-old male Cobb x Cobb 500, allocated into 80 battery cages with 8 chicks in each, were fed a low Cu content diet (formulated with 8.58 ± 0.21 mg/kg Cu) without phytase from placement to day 7. Starting on day 8, battery cages were distributed into a 2 × 5 factorial arrangement (phytase-added diets X 5 with graded increases of supplemental Cu) until day 28. Feeding treatments (feeds added or not with phytase and 5 graded increases of Cu) were randomly distributed with 8 cages of 8 chicks. The basal non-supplemented feeds were formulated with corn and soybean meal (SBM) without any other significant Cu contributors. Supplemental Cu was from laboratory-grade Cu sulfate pentahydrate (CuSO_5_H_2_0) which was increasingly added to the feeds. Phytase was added in excess to the producer recommendation (2,500 FYT) and had average analyzed values of 2,768 ± 135.2 FYT/kg whereas analyzed Cu values were: 8.05 ± 0.25, 11.25 ± 0.15, 14.20 ± 0.40, 16.55 ± 0.05, and 19.45 ± 0.45 mg/kg. Statistics were conducted using linear and quadratic polynomial regression models. No interactions occurred between dietary Cu and phytase (*p* > 0.05) for any response and no effects were found for the individual factors (phytase or dietary Cu) for Ht, Hb, varus, valgus, rotated tibia, and tibia breaking strength, as well as for Cu contents in breast, gastrocnemius tendon, and kidney (*p* > 0.05). However, the phytase-added diets led to higher BWG, lower FCR, and increased ileal digestible Cu (*p* < 0.05). The gradual increase in dietary Cu produced linear increases in Cu content in livers, as well as in excreta and retention (*p* < 0.05). Supplementing phytase at levels expected to maximize phytate degradation was demonstrated to improve BWG and FCR; however, no effects were observed when dietary Cu was increased to a maximum of 19.45 mg/kg. An increase of 8.8% in ileal digestible Cu was observed when birds were fed phytase.

## Introduction

Routine supplementation of dietary Cu, as well as other trace minerals, is intended to prevent deficiencies that originate from the lack of sufficient amounts of this micromineral in the feeds. Copper sulfate (CuSO_4_) is traditionally utilized as the source Cu of supplementation in poultry feeds because of its wide availability and low cost (1).

Total supplemental Cu in commercially formulated broiler feeds is variable, as can be observed in the recommendation tables, from as low as 6.5 in the NRC (2) to as high as 15 mg/kg (3) with others standing in between [10.5 mg/kg from Rostagno et al. (4) and 10.0 mg/kg from Aviagen (5)]. Since these are supplemental recommendations, Cu originating from feed ingredients, other than the supplemental source, is usually not taken into consideration for broilers. Copper has also been frequently added to broiler feeds at levels much higher than those suggested, intending to improve performance (6–9). The mode of action of such excessive dietary Cu supplementation is unknown, although it likely relates to antimicrobial properties (10).

Copper has several important functions in poultry, participating in bone formation, Fe transport, and in ceruloplasmin and cytochrome C oxidase and hemoglobin synthesis (11–14). Also, as a component of ferroxidase, Cu is important in the conversion of Fe+^2^ into Fe+^3^ (10, 15), which is required for Fe absorption and transport in animal organisms. Dietary Cu deficiency results in reduced blood hemoglobin (Hb), inducing anemia (16) and therefore blood parameters such as hematocrit (Ht) and Hb are frequently used to assess Cu bioavailability (17). As a cofactor of lysyl oxidase, Cu supports the formation of collagen and elastin and its deficiency leads to inadequate cartilage formation (18, 19). However, excess dietary Cu leads to hepatocellular degeneration, necrosis, or visual presence of Kupffer cells (20).

The published bioavailability of Cu for broilers varies from 80% in corn (21) to as low as 40% in soybean meal (SBM) (22). A major concern regarding the availability of Cu is related to its potential chelation to phytic acid (1,2,3,4,5,6 myo-inositol hexakis dihydrogen phosphate), a main storage form of P in plants that is widely distributed in feedstuffs (23–25). Phytic acid has a high density of negatively charged phosphate groups, therefore rendering it highly reactive with di- and trivalent cations. Commercial phytases have been widely added to poultry feeds with successful releases of significant amounts of P from phytic acid (26–30). Demonstrations of increased availability of trace minerals by phytase are much more limited than for Ca and P, even though increased ileal digestibility of Fe (31), Zn, Mn, and Cu have been recently shown (32).

Total Cu in excreta increases with its dietary intake (8, 10, 33, 34). Concerns about the excessive excretion of trace minerals and environmental pollution have been growing around the world, which has led to the establishment of maximum upper limits for total Cu in feeds of 25 ppm in the European Union (35).

The objective of the present study was to assess the availability of Cu for broilers when phytase was added to their feeds. Phytase was added at levels above current market usage such that any significant release of Cu could be detected under a potential extensive degradation of phytate. Supplementation of Cu was done using Cu sulfate such that feeds with values that mimic its contents in commercial corn-soy feeds were reached and therefore no limitations in growth could be attributed to its deficiency.

## Materials and methods

The Ethics and Research Committee of the Federal University of Rio Grande do Sul, Porto Alegre, Brazil, approved all procedures used in the present study (Protocol number: 40248).

### Bird husbandry and dietary treatments

A total of 640 one-day-old male Cobb ×Cobb 500 slow feathering broiler chicks were obtained after incubating eggs in the University hatchery and were allocated into 80 battery cages (experimental units) (0.9 × 0.4 m^2^). The chicks were vaccinated for Marek’s disease and feather sexed to keep only males. The temperature at placement was 32°C, which was adjusted weekly to maintain bird comfort throughout the study. The chicks had *ad libitum* access to water and mash feeds. Lighting was provided for 24 h continuously throughout the study. All battery pens were daily checked for sick and dead birds.

The birds were fed a diet of corn-SBM without phytase and not supplemented with Cu (8.58 ± 0.2 ppm analyzed) from day 1 to 7 (Table 1). This was a low Cu diet intending to reduce in Cu stored in the body. A random allocation of birds to treatments was in a 2 vs. 5 factorial: feeds with or without phytase and 5 graded increases of supplemental Cu. Therefore, a total of 10 treatments with 8 replications of 8 birds each were utilized. Birds were kept until 28 days of age. Dietary treatments consisted of corn-SBM feeds with energy and nutrients to optimize live performance as usually formulated in broiler integrations. Feeds were mixed in batches of 400 kg and provided as mash. The phytase utilized in the present study was a commercially available product added at 125 g per ton (Ronozyme HiPhos with 20,000 FYT/g, Novozymes A/S, Bagsvaerd, Denmark), expecting to deliver 2,500 FYT/kg (2,768 ± 135.2 analyzed). The phytase in the present study was added in excess of its commercial recommendation of 1,000 FYT, such that the vast majority of the phytate present in corn and SBM was overwhelmingly degraded. The product was added on top of the feeds without attribution of value for P and Ca in mixes of 1 kg diluted with SBM corresponding to the contents expected in the treatments. Supplementation of Cu was from laboratory-grade sulfate pentahydrate (CuSO_5_H_2_O) at 0, 3.0, 6.0, 9.0, and 12.0 mg/kg. Calcium carbonate and phosphoric acid were also laboratory-grade without significant Cu contents. The final formulated Cu in feeding treatments was 8.31, 11.31, 14.31, 17.31, and 20.31 mg/kg. The feeds provided from days 8 to 28 had the inclusion of 1% indigestible marker (as is, Celite, Celite Corp., Lompoc, CA) and had an average geometric diameter of 1.124 μm with a standard deviation of 1.79. The phytase activity in the feeds was measured according to Engelen et al. (36) and expressed in phytase units (FYT, defined as the activity that releases one μmol of inorganic phosphate from 5.0 mM sodium phytate/min at pH 5.5 and 37°C). Analyses of Cu in ingredients and feeds were performed using inductively coupled plasma atomic emission spectroscopy (ICP-Spectro Flamme, Spectro Analytical Instruments, Kleve, Germany) (37).

**Table 1:** Ingredient and nutrient composition of feeds formulated to evaluate Cu requirements of broilers having phytase.

	Low Cu pre-starter	Starter
	1 to 7 d	8 to 28 d
Ingredient, %^a^		
Corn, 7,8	48.50	47.04
Soybean meal, 46	45.47	41.69
Soybean oil	2.80	5.41
Calcium carbonate	1.55	2.23
Celite	-	1.00
Phosphoric acid	0.37	1.36
Salt	0.53	0.51
DL-Methionine, 99%	0.38	0.35
L-Lysine HCl, 76%	0.10	0.10
L-Threonine, 98.5%	0.08	0.07
Choline chloride	0.02	0.04
Vitamin and mineral mix^b^	0.20	0.20
Total	100.00	100.00
Calculated nutrient composition, % unless noted^c^		
AME_n_, kcal/kg	2,960	3,050
CP	24.9 (24.8 ± 0.24)	23.17 (22.9 ± 0.29)
Ca	1.05 (1.03 ± 0.03)	0.95 (0.93 ± 0.02)
P total	0.67 (0.65 ± 0.02)	0.65 (0.62 ± 0.02)
Non-phytate P	0.52	0.47
Phytate	0.23 (0.23 ± 0.02)	0.22 (0.22 ± 0.02)
Na	0.23	0.22
Choline, mg/kg	1,600	1,600
Dig. Lys	1.32	1.22
Dig. Met + Cys	0.99	0.91
Dig. Thr	0.86	0.79
Dig. Trp	0.28	0.26
Dig. Val	1.02	0.94
Phytase, FYT/Kg^d^	–	2,500
Cu, ppm^e^	8.63 (8.58 ± 0.21)	8.31

a Calcium carbonate and phosphoric acid were laboratory grade and had only trace amounts of Cu (2.1 and 0.3 mg/kg, respectively).

b Composition per kilogram of feed: vitamin A, 12,000 IU; vitamin D_3_, 3,000 IU; vitamin C, 50 mg; vitamin E, 100 IU; vitamin K_3_, 6 mg; vitamin B12, 35 μg; thiamin, 3 mg; riboflavin, 15 mg; vitamin B6, 6 mg; niacin, 40 mg; pantothenic acid, 25 mg; folic acid, 4 mg; biotin, 0.3 mg, Zn, 100 ppm; Mn, 100 ppm; Fe, 50 ppm; Se, 0.45 mg and I, 2 mg.

c Analyzed values are in the parentheses.

d Ronozyme HiPhos 20,000 FYT/g, Novozymes A/S, Bagsvaerd, Denmark; analyzed phytase in the Cu supplemented feeds were (from the lowest to the highest Cu content feeds) 2,752 ± 171; 2,864 ± 264; 2,831 ± 391; 2,746 ± 282 and 2,747 ± 149 FYT/kg.

e Formulated Cu in feeding treatments were 8.31, 11.31, 14.31, 17.31, 20.31 mg/kg; analyzed Cu in the feeds without phytase were 7.83 ± 0.2, 11.11 ± 0.4, 14.62 ± 0.1, 16.47 ± 0.2, 18.96 ± 0.1 whereas in the feeds with phytase were 8.28 ± 0.2, 11.36 ± 0.6, 13.77 ± 0.3, 16.63 ± 0.2, 19.94 ± 0.4.

### Growth performance, total excreta, and ileal contents

Body weight gain (BWG), feed intake (FI), and feed conversion ratio corrected for the weight of dead birds (FCR) were measured at the intervals between 8 to 14, 14 to 21, and 21 to 28 d as well as in the overall period (8 to 28 d). From 26 to 28 d, the total amount of feed consumed was recorded and the corresponding excreta were collected. Excreta were pooled by cage and stored frozen at −20°C immediately after collection in plastic containers. Ileal contents were collected from all birds from each cage at 28 d after euthanasia by electrical stunning using 45 V for 3 s. Contents were collected from the terminal two-thirds ileum, which was defined as the region between Meckel’s diverticulum to 2 cm cranial to the ileocecal junction. Contents were flushed with distilled water into plastic containers, pooled by battery cage, immediately frozen in liquid nitrogen, and stored in a freezer at −20°C until lyophilized. Feed samples and ileal contents were analyzed to determine dry matter (DM), gross energy (GE), and total Cu. Calculations of ileal digestible energy (IDE) were done afterward. Apparent ileal digestibility was calculated using the equations from Kong and Adeola (38) as follows: digestibility (%) = [1 – (M_i_/M_o_) × (E_o_/E_i_)] × 100, where M_i_ represents the concentration of acid insoluble ash in the feeds in grams per kilogram of DM; M_o_ represents the concentration of acid insoluble ash in the ileal digesta in grams per kilogram of DM output; E_i_ represents the concentration of DM, GE, Cu in the diet in milligrams per kilogram of DM; and E_o_ represents the concentration of DM, GE, Cu, in the ileal digesta in milligrams per kilogram of DM.

### Leg deviations

At 26 d, *valgus*, *varus,* and rotated tibiae deviations at the tibia metatarsal joints were evaluated in all birds standing in the normal anatomic position by a 3 individual panel, classifying them by the presence or the absence of each deviation. Tibiae-metatarsal joints with valgus deviations were those presenting an outward angulation, whereas the varus deviations were inward (39). Rotated tibiae above 90° were considered abnormal and characterized as a torsional rotation of the shaft of the tibiotarsus of 1 or both legs, causing the metatarsus to point laterally and the broiler to assume a spraddle leg posture (40). Scores taken were averaged for the sake of statistical analysis.

### Blood and tissue sampling

Blood samples were taken by heart punctures prior to slaughter from three broilers randomly selected from each cage. The obtained blood was partially transferred to 0.5 mL test tubes containing EDTA for hematocrit (Ht) and hemoglobin (Hb) analyses. Determination of Ht was done using micro capillaries containing blood centrifuged for 5 min at 15,650–18,510×*g*, whereas Hb was determined using the cyanmethemoglobin method as described by Crosby et al. (41).

Breast muscle (*Pectoralys major*), liver, kidney, and gastrocnemius tendon samples were collected from all birds after euthanasia at 28 d. Collected samples were weighed and stored in plastic bags by pen and remained at −20°C until analysis. Samples were further ashed and Cu content was determined as done with the feeds. The left tibiae of all birds from each cage were collected and the surrounding muscle tissue was removed. Samples were frozen until analysis. Tibia breaking strength was evaluated using a texture analyzer (TA.XTPlus, Texture Technologies, Hamilton, MA, United States), according to the method proposed by Shim et al. (42).

### Statistical analysis

Data were tested for homoscedasticity and normality of the variance prior to statistical analyses (43). Data that were not normally distributed were square root transformed prior to analyses; however, real means are presented as results. A two-way ANOVA was used to test for differences in the effects of the independent variables (phytase and dietary Cu) on the dependent variables measured. Analyses were done using the GLM procedure of SAS (44). Significance was accepted at *p* ≤ 0.05. Data were submitted to a two-way ANOVA and mean differences were separated using Tukey’s HSD test.

Estimation of optimum responses to total dietary Cu was done using linear (L) and quadratic polynomial (QP) regression analyses. The L model (*Y* = *β*1 + *β*2 × *X*) had *Y* as the dependent variable, *X* as the dietary concentration of Cu, *β*1 as the intercept, and *β*2 as the linear coefficient. The QP model [*Y* = *β*1 + *β*2 × Cu + *β*3 × (Cu)^2^] had *Y* as the dependent variable as a function of the dietary concentration of Cu, *β*1 as the intercept, *β*2 as the linear coefficient, and *β*3 as the quadratic coefficient. The maximum response for Cu concentration would be found as Cu = −*β*2 ÷ (2 × *β*3).

## Results

The analyzed Cu in the experimental feeds was similar to that expected from the feed formulation (Table 1) and, therefore, the feeds were considered acceptable for the experimental assessment originally planned. The final formulated Cu in feeding treatments were 8.31, 11.31, 14.31, 17.31, 20.31 mg/kg (analyzed Cu in the feeds without phytase were 7.83 ± 0.2, 11.11 ± 0.4, 14.62 ± 0.1, 16.47 ± 0.2, 18.96 ± 0.1, whereas in the feeds with phytase were 8.28 ± 0.2, 11.36 ± 0.6, 13.77 ± 0.3, 16.63 ± 0.2, 19.94 ± 0.4). The final analyzed phytase in the Cu-supplemented feeds was (from the lowest to the highest Cu content feeds) 2,752 ± 171; 2,864 ± 264; 2,831 ± 391; 2,746 ± 282; and 2,747 ± 149 FYT/kg.

Two-way ANOVA and regression analyses were conducted with the Cu-analyzed data whenever no interaction was found.

There was no mortality and no individuals were detected as sick during the study. No interactions between dietary Cu and phytase were found for any evaluated response throughout the study. Individual factors (phytase and Cu) did not affect Ht, Hb, varus, valgus, rotated tibia, tibia breaking strength, or Cu concentration in breast, gastrocnemius tendon, and kidney (*p* > 0.05; Table 2). However, broilers fed phytase showed higher BWG and lower FCR, and higher FI (*p* < 0.01; Table 3), IDE, and ileal digestibility of Cu (*p* < 0.05; Table 4) when compared to broilers that had no phytase supplementation. Responses to Cu supplementation were only observed for Cu intake, excretion, liver content, and retention. These responses were linearly correlated to the increasing dietary Cu as follows: Cu intake = 2.3618 + 0.8507*x*, *R*^2^ = 0.90, *p* < 0.001; Cu excreted = 0.2605 + 0.2814*x*, *R*^2^ = 0.82, *p* < 0.001; 1iver Cu content = 2.3617 + 0.85076*x*, *R*^2^ = 0.73, *p* < 0.001; and Cu retained = 0.2645 + 0.6849*x*, *R*^2^ = 0.80, *p* < 0.001. There was no response that could be quadratically adjusted, which prevented estimations of optimum dietary levels of Cu.

**Table 2:** Blood parameters, leg deviations, tibia breaking strength, and Cu content in tissues of broilers fed diets formulated to evaluate the Cu requirements of broilers receiving phytase.

Treatment^1^	Ht, %^2^	Hb, g/dL	Leg deviations, %^5^				Tibia breaking strength, *N*	Breast muscle	Gastrocnemius tendon	Kidney	Liver^6^
			Unaffected	Valgus	Varus	Rotated tibia		Cu, mg/kg			
Phytase, FYT/kg^3^											
None	27.1	8.22	48.7	45.4	2.5	2.4	300	1.44	1.97	11.3	10.71
2,500	27.5	8.25	49.6	47.3	1.9	2.2	315	1.41	1.85	11.1	10.76
Cu, mg/kg^4^											
8.31	26.4	8.38	50.2	44.6	2.5	2.7	308	1.40	1.91	11.3	9.80^b^
11.31	27.1	8.09	49.2	47.9	1.6	1.3	294	1.49	1.72	12.3	10.01^b^
14.31	27.5	8.26	48.4	45.0	2.8	3.8	318	1.38	2.02	11.1	10.30^ab^
17.31	27.3	8.24	47.8	48.1	2.0	2.1	321	1.39	2.00	10.6	11.78ª
20.31	28.0	8.20	50.0	46.1	2.1	1.8	300	1.47	1.87	10.8	11.82^a^
SEM	0.197	0.068	1.007	1.029	0.418	0.481	5.249	0.028	0.073	0.220	0.253
*Probability*											
Phytase	0.442	0.806	0.675	0.769	0.256	0.690	0.161	0.498	0.411	0.702	0.876
Cu	0.129	0.772	0.934	0.343	0.822	0.443	0.446	0.688	0.665	0.125	0.001
Phytase × Cu	0.091	0.456	0.552	0.317	0.063	0.519	0.574	0.289	0.204	0.511	0.204

^a,b^Means within the same column with different superscripts differ by Tukey test (*p* ≤ 0.05).

1 Replications per treatment: *n* = 8.

2 Ht, Hematocrit; Hb, Hemoglobin.

3 Ronozyme HiPhos 20,000 FYT/g, Novozymes A/S, Bagsvaerd, Denmark; analyzed phytase in the Cu supplemented feeds were (from the lowest to the highest Cu content feeds) 2,752 ± 171; 2,864 ± 264; 2,831 ± 391; 2,746 ± 282 and 2,747 ± 149 FYT/kg.

4 Formulated Cu in feeding treatments were 8.31, 11.31, 14.31, 17.31, 20.31 mg/kg; analyzed Cu in the feeds without phytase were 7.83 ± 0.2, 11.11 ± 0.4, 14.62 ± 0.1, 16.47 ± 0.2, 18.96 ± 0.1 whereas in the feeds with phytase were 8.28 ± 0.2, 11.36 ± 0.6, 13.77 ± 0.3, 16.63 ± 0.2, 19.94 ± 0.4.

5 Probabilities of deviations at the tibiae metatarsal joints presented after arcsine transformation.

6 Liver Cu content = 2.3617 + 0.85076X, *R*^2^ = 0.7253, *p* < 0.001; X, dietary Cu, mg/kg.

**Table 3:** Growth performance of broilers fed diets formulated to evaluate the Cu requirements of broilers receiving phytase.

Treatment^1^	8 to 28 d^2^		
	BWG, g	FCR	FI, g
Phytase, FYT/kg^3^			
None	1,384	1.301	1,807
2,500	1,453	1.286	1,868
Cu, mg/kg^4^			
8.31	1,405	1.299	1,828
11.31	1,434	1.289	1,854
14.31	1,433	1.293	1,851
17.31	1,419	1.288	1,829
20.31	1,404	1.300	1,825
SEM	6.825	0.003	7.487
*Probability*			
Phytase	0.001	0.005	0.001
Cu	0.261	0.448	0.478
Phytase × Cu	0.592	0.061	0.344

Means within the same column with different superscripts differ by Tukey test (*p* ≤ 0.05).

1 Replications per treatment: *n* = 8.

2 BWG, body weight gain; FCR, feed conversion ratio corrected for the weight of dead birds; FI, feed intake.

3 Ronozyme HiPhos 20,000 FYT/g, Novozymes A/S, Bagsvaerd, Denmark; analyzed phytase in the Cu supplemented feeds were (from the lowest to the highest Cu content feeds) 2,752 ± 171; 2,864 ± 264; 2,831 ± 391; 2,746 ± 282 and 2,747 ± 149 FYT/kg.

4 Formulated Cu in feeding treatments were 8.31, 11.31, 14.31, 17.31, 20.31 mg/kg; analyzed Cu in the feeds without phytase were 7.83 ± 0.2, 11.11 ± 0.4, 14.62 ± 0.1, 16.47 ± 0.2, 18.96 ± 0.1 whereas in the feeds with phytase were 8.28 ± 0.2, 11.36 ± 0.6, 13.77 ± 0.3, 16.63 ± 0.2, 19.94 ± 0.4.

**Table 4:** Metabolism responses of broilers fed diets formulated to evaluate Cu requirements (dry matter basis)^1^ when receiving phytase.

Item	Intake	Excreta	Cu Retained^4^		Ileal	
	Cu, mg/bird		mg/bird	%	Coefficient of digestibility of Cu, %	Digestible energy, kcal/kg
Phytase, FYT/kg^2^						
None	14.13	4.26	9.87	70.3	31.8	2,675
2,500	13.85	4.13	9.72	69.3	34.6	2,881
Cu, mg/kg^3^						
8.31	8.22^e^	2.50^e^	5.72^d^	69.1	30.6	2,636
11.31	11.34^d^	3.41^d^	7.93^c^	69.7	34.0	2,741
14.31	14.79^c^	4.27^c^	10.30^b^	70.9	34.1	2,721
17.31	16.75^b^	5.09^b^	11.66^b^	69.4	33.7	2,926
20.31	19.08^a^	5.68^a^	13.40^a^	70.0	33.8	2,866
SEM	0.461	0.141	0.347	0.527	1.081	23.99
*Probability*						
Phytase	0.594	0.276	0.602	0.367	0.013	0.037
Cu	0.001	0.001	0.001	0.856	0.238	0.281
Phytase × Cu	0.298	0.060	0.191	0.059	0.171	0.985

^a,b^Means within the same column with different superscripts differ by Tukey test (*p* ≤ 0.05).

1 Intake, excreta, and retention of Cu (mg/kg), respectively: *Y* = 2.3618 + 0.8508*x*, *R*^2^ = 0.9081, *p* < 0.001; *Y* = 0.2605 + 0.2814*x*, *R*^2^ = 0.8158, *p* < 0.001; *Y* = 0.2645 + 0.6849*x*, *R*^2^ = 0.8020, *p* < 0.001; *X* = dietary Cu content.

2 Ronozyme HiPhos 20,000 FYT/g, Novozymes A/S, Bagsvaerd, Denmark; analyzed phytase in the Cu supplemented feeds were (from the lowest to the highest Cu content feeds) 2,752 ± 171; 2,864 ± 264; 2,831 ± 391; 2,746 ± 282 and 2,747 ± 149 FYT/kg.

3 Formulated Cu in feeding treatments were 8.31, 11.31, 14.31, 17.31, 20.31 mg/kg; analyzed Cu in the feeds without phytase were 7.83 ± 0.2, 11.11 ± 0.4, 14.62 ± 0.1, 16.47 ± 0.2, 18.96 ± 0.1 whereas in the feeds with phytase were 8.28 ± 0.2, 11.36 ± 0.6, 13.77 ± 0.3, 16.63 ± 0.2, 19.94 ± 0.4.

4 Probabilities of Cu retained and coefficient of digestibility Cu presented after arcsine transformation.

## Discussion

The content of Cu in feedstuffs varies depending on its original area of production (2, 45). Copper has been supplemented to broiler feeds because of uncertainty about its availability in plant feedstuffs and, therefore, to avoid deficiency. Nonetheless, poultry excreta has high Cu regardless of the level utilized in feeds, which eventually leads to increased Cu in soils used for agriculture (8, 46, 47). In the present study, diets fed to broilers without Cu supplementation optimized broiler growth up to 28 d. In parallel, no effects were observed for other responses not directly related to growth, regardless of the addition of phytase. Therefore, the recommendations from the NRC (2), Cobb (3), Rostagno et al. (4), and Aviagen (5) were considered excessive whereas the limit of 25 mg/kg imposed by EFSA (35) is well above broiler needs.

The addition of exogenous phytase in poultry feeds is commercially mandatory currently since it significantly increases P availability from plant feedstuffs, leading to the reduction in feed costs due to a lower inclusion of phosphate sources. The increased availability of Ca, protein, amino acids, and other minerals than P has been shown as a secondary benefit from the use of phytases with a consequent reduction in excreta (48–52). In the present study, broilers fed phytase showed higher BWG and lower FCR when compared to those that were not fed the enzyme. Phytase was added on top of the feeds without attributing any P or Ca values in the formulation, therefore, the improvements were thought to have other origins. The contents of non-phytate P (nPP) and Ca in the feed formulations in the present study were as is usual in commercial integrations and similar to popular allowance recommendations [0.45% nPP and 1.00% Ca from the NRC (2); 0.43% nPP and 95% Ca from FEDNA (53); 0.43% nPP and 0.91% Ca from Rostagno et al. (4); 0.42% nPP and 0.84% Ca from Cobb (3)]. Apart from Cu, all other trace minerals were supplemented. Therefore, Cu was the only trace mineral not supplemented and the benefits of phytase regarding the further availability of Cu could be assessed. Phytase additions in feeds in amounts that surpass optimized phytate degradation and capability to provide P from phytate (super doses) have sometimes been reported as extra phosphoric effects with improvements in the availability of energy, amino acids, and in the performance of broilers (54–59).

Hematological analyses can be efficient clinical tools in the diagnosis of metabolic disorders. Although Cu is not a constituent of hemoglobin itself, Cu is an active participant in the oxygen transport process as it is present in certain plasma proteins that are involved in the transport of Fe (12, 60). When varying levels of dietary Cu were evaluated with broilers and broiler breeders, hemoglobin was markedly lower than in those supplemented with Cu (9, 61). In the present study, there were no differences in the measured blood parameters which suggests that Cu in the non-supplemented feeds had sufficient Cu to maximize those measurements properly inducing red-blood-cell formation. Interestingly, it has been earlier suggested by Hill and Matrone (62) that Cu deficiency alone has little effect on hemoglobin concentration and hematocrit values.

Proper bone mineralization demands Cu, which is ultimately derived from the feed (63, 64). Locomotor limitations are currently important morbidity signals and, therefore, indicators of the welfare of broiler chickens (35, 65). In the present experiment, there were no effects of Cu supplementation in the frequency of varus or valgus deviations, or in tibia rotation due to dietary Cu. Day-old chicks fed Cu deficient feeds with increasing levels of Cu for 21 days showed optimum growth when fed 6–8 mg/kg Cu whereas the requirement for normal cross-link formation and bone mineralization was lower than 2 mg/kg Cu (19). The lack of Cu supplementation effects in the gastrocnemius tendon paralleled the outcomes in bone ash in the present study. This tendon is a sensitive indicator of Cu deficiency (66). Thus, it seems that increasing Cu in corn-SBM feeds through Cu sulfate supplementation does not impact the most prevalent locomotor capabilities of broilers.

According to the present results, there were differences among treatments in liver Cu contents when measured on day 28. Previous studies have reported increased Cu in the liver along with its increase in feeds (7, 13, 67–70). Despite the wide range of dietary Cu concentrations fed to poultry in different commercial settings, its proportional increase in livers seemed not to reach a concentration capable of producing signals of toxicity (71, 72). The increase in Cu excretion as well as in body retention that paralleled liver contents in the present study have been earlier noticed by others (33, 73, 74). The frequent ultimate disposal of poultry excreta as a soil amendment will certainly lead to a steady increase of Cu in the environment. Moreover, the linear excretion of Cu that parallels its content in the feeds is an explicit indicator of its dietary excess when supplemented further than what is provided by commercial corn-SBM feeds.

Broiler feeds supplemented with phytase in a corn-SBM diet presented higher IDE in the present study. Leyva-Jimenez et al. (75) reported that phytase added at 1,000 FYT/Kg led to increased IDE in 24-day-old broilers and Walters et al. (76) observed higher IDE when broilers were fed phytase at 1,000 and 2,000 FTY/kg. Supplementation with phytase allows a reduction of endogenous losses in the gastrointestinal tract, allowing an increase in metabolizable energy and a reduction in energy needed for maintenance. This allows for a greater amount of energy for growth (77). The benefits of using phytase at levels higher than 1,000 FYT have been related to higher absorption of myo-inositol with a parallel increase in the retention of dietary energy (48).

In the present study, the phytase addition led to a higher Cu ileal digestibility when compared to broilers that were not fed the enzyme. To the knowledge of this study’s authors, the benefits of phytase on Cu availability have only been reported in pigs (78). The increase in the ileal digestibility of Cu occurred regardless of the level of Cu in the feeds and, therefore, this seems to have resulted from the release of Cu chelated to phytate. The increased coefficient of digestibility of Cu when broilers were fed a high dose of phytase was small; however, this effect was obtained without assessing phytate degradation directly.

## Conclusion

Adding phytase at a dose expected to degrade the vast majority of phytate present in broiler feeds leads to growth performance improvements, which seems to be related to the increased utilization of dietary energy. The effect of phytase on Cu digestibility is significant, but small in value when the total Cu requirement is considered. Increases in Cu supplementation to levels that mimic the total provision of this mineral in commercial feeds have no benefit in responses related to growth or blood, or in the most common locomotor problems. Excess dietary Cu leads to its increased content in the liver as well as in excreta, which may lead to an eventual environmental burden.

## Data availability statement

The raw data supporting the conclusions of this article will be made available by the authors, without undue reservation.

## Ethics statement

The animal studies were approved by Comissão de Ética no Uso de Animais—UFRGS. The studies were conducted in accordance with the local legislation and institutional requirements. Written informed consent was obtained from the owners for the participation of their animals in this study.

## Author contributions

PS: research and analysis. SV: conceptualization and research. JF: research, writing, and analysis. WA: research. GT: research. All authors contributed to the article and approved the submitted version.

## Funding

Fundação de Apoio da Universidade Federal do Rio Grande do Sul.

## Conflict of interest

The authors declare that the research was conducted in the absence of any commercial or financial relationships that could be construed as a potential conflict of interest.

## Publisher’s note

All claims expressed in this article are solely those of the authors and do not necessarily represent those of their affiliated organizations, or those of the publisher, the editors and the reviewers. Any product that may be evaluated in this article, or claim that may be made by its manufacturer, is not guaranteed or endorsed by the publisher.
